# Chicago College of Pharmacy

**Published:** 1860-01

**Authors:** 


CHICAGO COLLEGE OF PHARMACY.—It is highly gratifying to the friends of progress and humanity to find that a college of pharmacy has recently been established in this flourishing city of the great Northwest. It is impossible, in view of the astonishing ignorance and carelessness of American apothecaries and druggists, that there should be too many institutions of this kind in the United States. We hope the day is not distant when every medicine dispenser shall be compelled to obtain a thorough professional education before he is permitted to enter upon his responsible avocation.

The Chicago College is to have a session of twenty weeks, a lecture being delivered every other day. The members of the faculty are James V. Z. Blaney, M.D., Professor of Chemistry; F. Scammon, M.D., Professor of Pharmacy; and John H. Rauch, M.D., Professor of Materia Medica.

